# Caribbean deepwater snappers: Application of the bomb radiocarbon age estimation validation in understanding aspects of ecology and life history

**DOI:** 10.1371/journal.pone.0295650

**Published:** 2023-12-27

**Authors:** Katherine E. Overly, Virginia R. Shervette

**Affiliations:** 1 Technical and Engineering Support Alliance, National Oceanic and Atmospheric Administration, National Marine Fisheries Service, Southeast Fisheries Science Center, Panama City, Florida, United States of America; 2 School of Forest, Fisheries, and Geomatics Sciences, University of Florida, Gainesville, Florida, United States of America; 3 Fish/Fisheries Conservation Lab, University of South Carolina Aiken, Aiken, South Carolina, United States of America; COISPA Tecnologia & Ricerca - Stazione Sperimentale per lo Studio delle Risorse del Mare, ITALY

## Abstract

Reef fishes have been utilized as food fish throughout the U.S. Caribbean and Gulf of Mexico waters for centuries, with increasing fishing effort in recent decades. As a result, many species have experienced declines in landings, including deepwater snappers such as queen snapper *Etelis oculatus* and blackfin snapper *Lutjanus buccanella*. However, little to no peer-reviewed published research exists on basic life history parameters for either species. Confirming the accuracy of an age estimation method for a fish species is essential for ensuring sustainable fisheries management. This is because in the assessment of fisheries species population age-based parameters, including longevity, age at sexual maturity, growth rate, mortality, age-specific reproductive output, and lifetime reproductive output, are important in understanding overall life history strategies of managed stocks. The past stock assessment on U.S. Caribbean queen snapper utilized an estimated longevity of 8 y, derived from length frequencies for fish from St. Lucia. Blackfin snapper has an estimated longevity of 27 y based on a relatively small study from offshore waters of the southeastern U.S. The focus of our investigation was to estimate maximum longevity of two data-poor species in the U.S. Caribbean. The accuracy of ageing methods was tested via bomb radiocarbon age estimation validation and effects of depth on Δ^14^C in otolith cores and eye lens core values were examined. Results from our work indicate a maximum validated age of 45 y for queen snapper, and 43 y for blackfin snapper. Our findings indicate queen snapper and blackfin snapper are long-lived (> 40 y). The resulting Δ^14^C comparison between eye lens cores and otolith cores has important implications for the study of age validation, specifically when deepwater species are involved.

## Introduction

Fisheries management in marine waters of the United States (U.S.) relies on a complex and scientifically rigorous stock assessment process, which results in management strategies that facilitate the long-term sustainable harvest of fisheries species. In the U.S. Caribbean and Gulf of Mexico (GoM) “deepwater snappers” is a general grouping used to reference snapper species landed in commercial and recreational fisheries, most commonly as adults, from deeper continental shelf waters to the slope (depths ranging from 100–500 m) [1, 2]. Little to no published life history data currently exists for these deepwater snapper species yet they support fisheries throughout the U.S. Caribbean and GoM regions. To conduct even the most basic of stock assessments for a species, life history information on population age structure, growth, longevity and natural mortality is required.

Age estimation of individuals within a fish population is a critical first step in documenting life history parameters. Despite their importance, age and growth studies are severely lacking for many U.S. Caribbean and GoM deepwater snapper species, including queen snapper *Etelis oculatus* and blackfin snapper *Lutjanus buccanella*. The primary method for estimating ages in deepwater snappers is by enumerating increments, alternating translucent and opaque growth zones, in transverse sections of sagittal otoliths [3–7]. However, environmental constancy in deepwater habitats can influence the clarity of otolith increment formation, which can negatively affect the accuracy of age estimation for deepwater snapper species. Therefore, validation of ageing methods is a critical first step in ensuring accurate age estimates are obtained [8, 9] and utilized for computing life history parameters.

Bomb radiocarbon (^14^C) analysis is a useful tool in the validation of age estimation methods for marine fishes [10–13]. Testing of nuclear weapons during the 1950’s and 1960’s caused a rapid increase in atmospheric ^14^C that was subsequently absorbed through air-sea diffusion into oceanic surface waters. The marine temporal record of the rapid increase and decline of bomb ^14^C (reported as Δ^14^C in the literature) in marine surface waters has been documented for various oceanic regions through analysis of biogenic CaCO_3_ from hermatypic corals [14–17] and fish otoliths [10, 13]. The CaCO_3_ in fish otoliths is inert once formed and thus records the Δ^14^C signal of the water where a fish resided when the otolith material formed [12, 18]. “Birth year” Δ^14^C values obtained from otolith cores (the region of the otolith that formed during the first year of life) can be compared with a regional Δ^14^C reference time series to validate the accuracy of fish age estimation [10, 13, 18–20].

Regional Δ^14^C reference time series reflect the temporal trends of the well mixed, epipelagic zone (< 200 m) in low- to mid-latitude oceans, below which ^14^C concentrations decrease with increasing depth [14, 21, 22]. Fish otoliths are composed primarily of aragonite (an inorganic form of CaCO_3_) and are metabolically inert once formed; the Δ ^14^C incorporated into aragonite of marine organisms is derived from dissolved inorganic carbon (DIC) of the surrounding water and, to a lesser extent, chemical constituents from food sources [23]. Therefore, fish as juveniles must live in and experience a similar water mass defined by the reference Δ^14^C time series [14] if otoliths are assumed to reflect the shallow water Δ^14^C values. Blackfin snapper in the Caribbean exhibit an ontogenetic transition in habitat depth; juveniles occur commonly in shallow reef habitat, while adults move to deeper waters with age [24, 25]. The depth of habitats utilized by queen snapper juveniles and adults are not well documented. Queen snapper larvae occur at depths of 0–100 m within the first two months of life, with 38-day old larva found as deep as 100 m [26]. Information on juvenile queen snapper is sparse, however a few juveniles ranging in size from 55 to 70 mm were recorded at a depth of 490 m [27], indicating that smaller, younger individuals are not restricted to shallow depths. Queen snapper adults regularly inhabit depths deeper than 200 m. The decreased flux of Δ^14^C to deeper continental shelf and slope waters in which queen snapper juveniles and adults reside could compromise the utility of using otolith core Δ^14^C to validate age estimation in this species [28].

Fish eye lens cores have recently been used as an alternative source of birth year Δ^14^C values for age estimation validation [29–32]. Eye lenses of fishes are similar to otoliths in that they continue to grow throughout the life of a fish and eye lens tissue is metabolically inert once formed [33, 34]. Eye lens tissue contains ~50% organic C by mass. Given this, Δ^14^C in eye lens cores can be utilized to validate age estimates of marine fishes, even if the lens cores are relatively small [31]. As relates to Δ^14^C age estimation validation of deepwater fish species, eye lens cores may provide a better source of birth year Δ^14^C compared to otolith cores. The organic carbon Δ^14^C of deep oceanic organisms is predominately derived from well-mixed euphotic zone phytoplankton sources [35, 36], thus eye lens cores from deepwater fish species would reflect a similar Δ^14^C as the reference time series established from shallow water carbonate sources [13, 15]. In contrast, as previously noted, otolith cores Δ^14^C would reflect the ^14^C of the DIC from deeper water if the fish resides there as a juvenile.

The overall goal of this work is to evaluate the usefulness of utilizing eye lens cores to validate age estimation of two deepwater snapper species. The specific objectives were to: (i) utilize eye lens cores to validate age estimation for queen snapper and blackfin snapper, (ii) update longevity estimates for the two species, and (iii) compare eye lens core Δ^14^C results to otolith core Δ^14^C results. A secondary goal was to summarize the relationships between depth of sample collection and fish size/age to examine potential ontogenetic shifts in habitat use for both species.

## Methods

This study was carried out in strict accordance with the recommendations in the Guide for the Care and Use of Laboratory Animals of the National Institutes of Health. The protocol was approved by the University of South Carolina Aiken Institutional Animal Care and Use Committee (Protocol Number: 053012-BIO-04).

### Fish collection and processing

**Fig 1:**
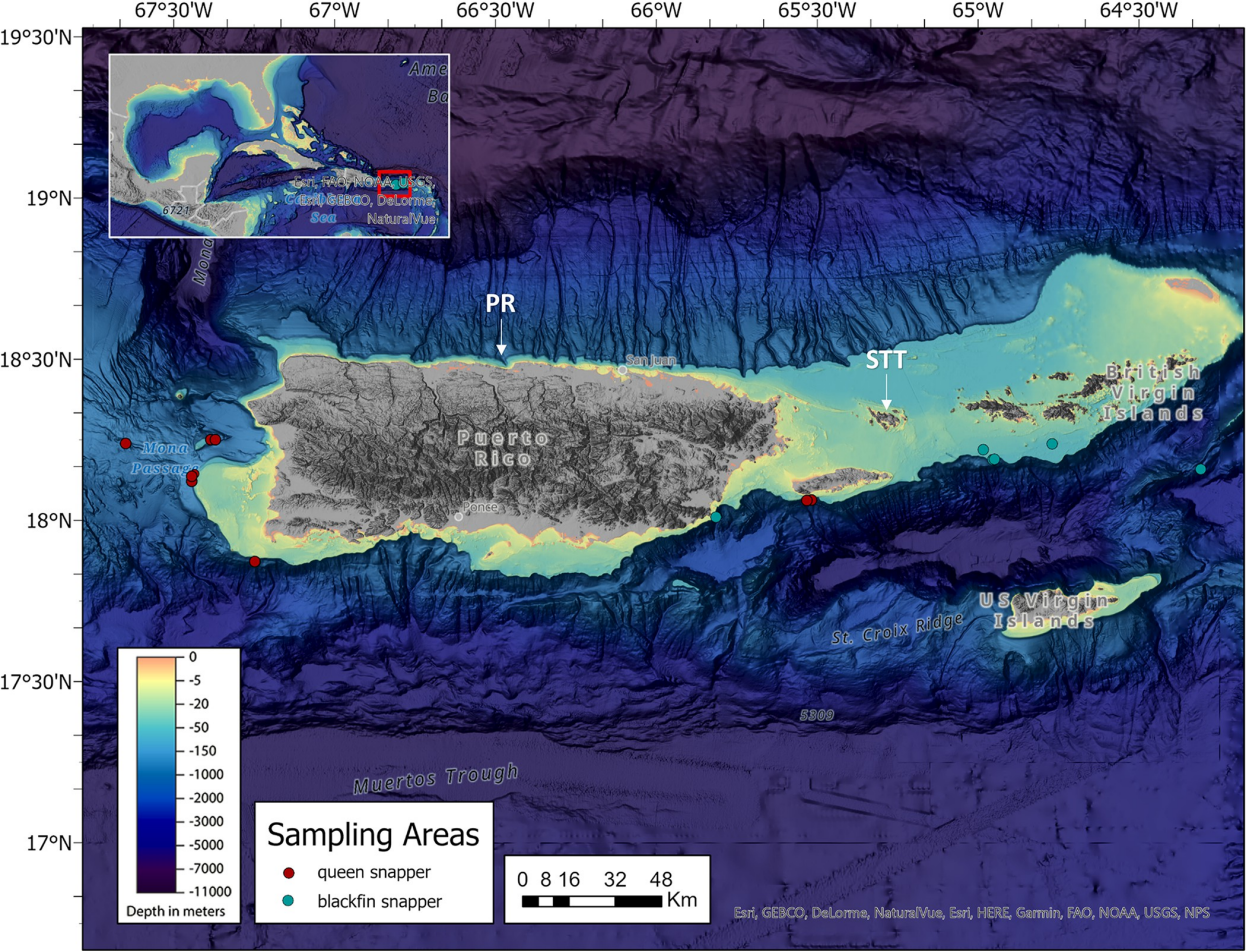
North Caribbean sampling region for queen and blackfin snapper. Map indicates the general north Caribbean region including the major islands of the U.S. Caribbean. General collections areas for blackfin snapper are indicated with teal circles and areas for queen snapper are indicated with red circles. The map layer used to generate this figure is from NOAA National Centers for Environmental Information and provided without restriction by the U.S. Government.

Queen and blackfin snapper samples were collected from waters of the U.S. Caribbean (Fig 1) through fishery-dependent (FD) and fishery-independent (FI) sampling programs. FD samples were obtained directly from fishers as they returned to shore. FI samples were obtained from NOAA Fisheries Puerto Rico Deepwater Reef Fish Survey (PR DWRFS) and the Southeast Area Monitoring and Assessment Program–Caribbean (SEAMAP-C). All fish were kept on ice until processing occurred. For each fish sample, date of collection, gear type used, and the general location of capture were recorded. For FI samples, depth and precise location of capture were also noted. Fish samples were measured for standard length (SL, mm), fork length (FL, mm), total length (TL, mm) and whole weight in kilograms (kg). Whole eyes were dissected from each FD sample, wrapped in foil, and stored at -20° C until further processing. Sagittal otolith pairs were extracted from all samples, wiped clean of tissue and debris, and stored dry for processing in the lab.

Fishery-independent sampling via the PR DWRFS was conducted by the NOAA Fisheries Panama City Laboratory using contracted commercial fishers. All FI fishing protocols were standardized between sampling areas and fishers. Sampling was conducted on the W/NW coast of PR at two hundred sampling sites following a stratified random statistical survey design by depth (100–500 m) and rugosity scores. Vertical hook and line fishing gear consisted of monofilament and spectrum line rigged with a 4.5-kg weight attached to the terminal end of the line, spooled on an electronic reel. Attached to the vertical main line were 12 leaders with 9/0 Mustad Extra Wide circle hooks baited with either California squid or mackerel. The gear was fished for 20 minutes after the weight reached the seafloor. Upon retrieval, all fish were measured immediately and kept on ice until processing occurred. Additional FI samples were collected for blackfin snapper through SEAMAP-C at depths ranging from 90–450 m via methods outlined in Cass-Calay et al. [37], and from 50–100 m depths via methods outlined in Gold and Portnoy [38]. Data from these collections were used to examine depth distribution as relates to fish length.

To examine the relationship among larval queen snapper size, daily age, and depth distribution, additional FI data was utilized from D’Alessandro et al. [26]. Samples were collected from the Straits of Florida (SOF) during daylight hours from January 2003 through December 2004 using a multiple opening-closing net and environmental sampling system (MOCNESS). Tows occurred in 25 m depth bins from surface water to 100 m, with a neuston net sampling the top ~0.5 m.

### Otolith preparation and age estimation

**Fig 2:**
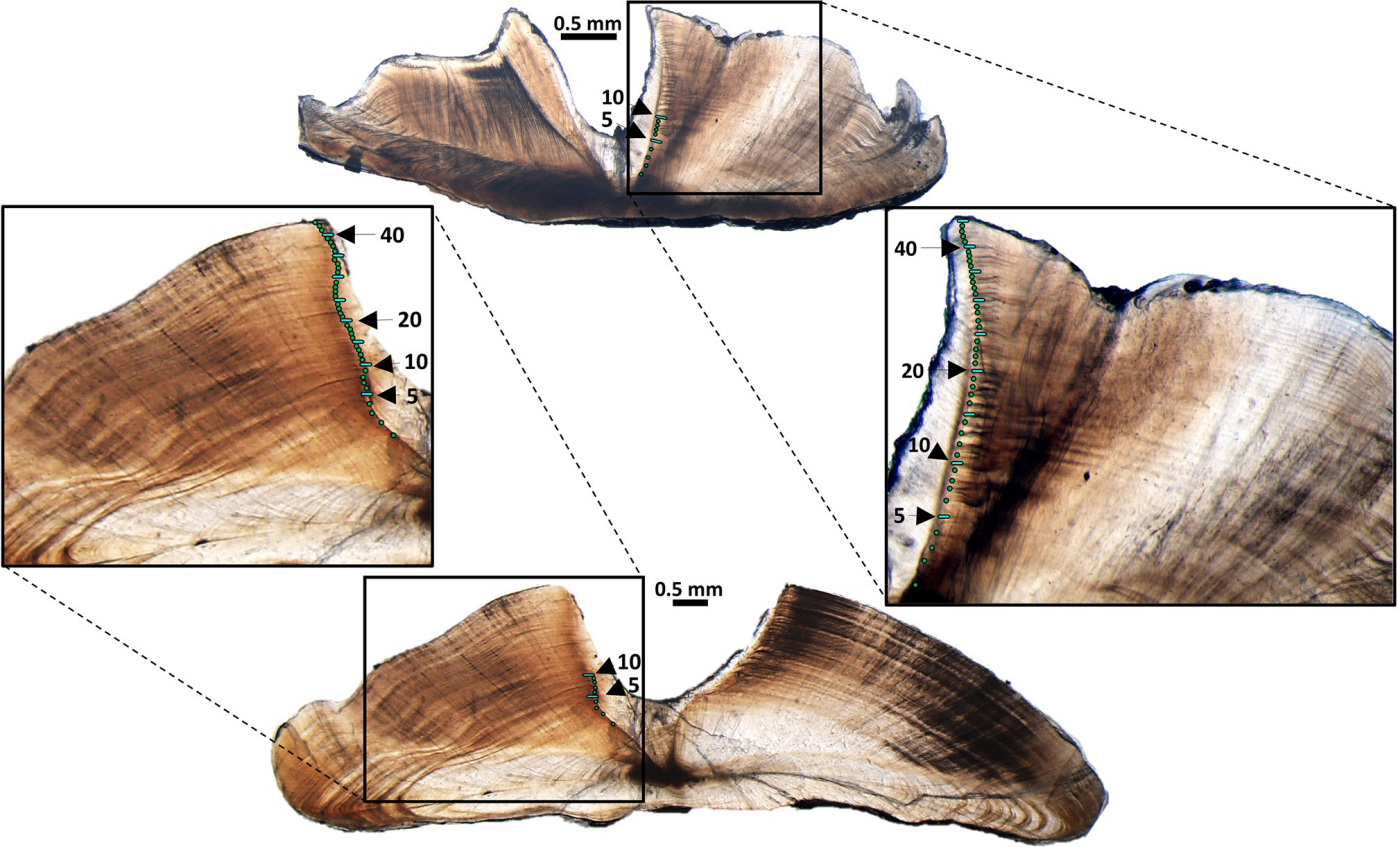
Annotated sagittal otolith sections for queen snapper and blackfin snapper. Examples of sagittal otolith sections for queen snapper (top) and blackfin snapper (bottom). Green circles denote the opaque zones observed on the otolith sections, whereas thick blue dashes denote every five opaque zones. Scale bars represent 0.5 mm. Reprinted under a CC BY license, with permission from Katherine Overly and Virginia Shervette, original copyright 2022.

To generate initial age estimates for queen and blackfin snappers, one otolith from each fish was embedded in epoxy. The left otolith was processed for ageing; however, the right otolith was used if the left was unavailable. A minimum of two thin transverse sections (0.3 mm thickness for queen snapper otoliths; 0.5 mm for blackfin snapper otoliths) were cut from the embedded otolith through the core region using a low-speed isomet saw equipped with a diamond-edged blade [13]. Otolith sections were fixed to microscope slides with clear mounting medium and read using transmitted light at a magnification of 40–50 x for queen snapper and a magnification of 15–25 x for blackfin snapper. The higher magnification for queen snapper was necessitated as the otoliths are small. Two independent readers recorded the number of opaque zones visible in otoliths sections for each fish sample (one pair of alternating translucent and opaque zones equals one increment; Fig 2). If disagreement between readers occurred, then the otolith sections were re-evaluated by both readers at the same time and a consensus opaque zone count was obtained as the final age estimate.

### Otolith and eye lens processing

**Fig 3:**
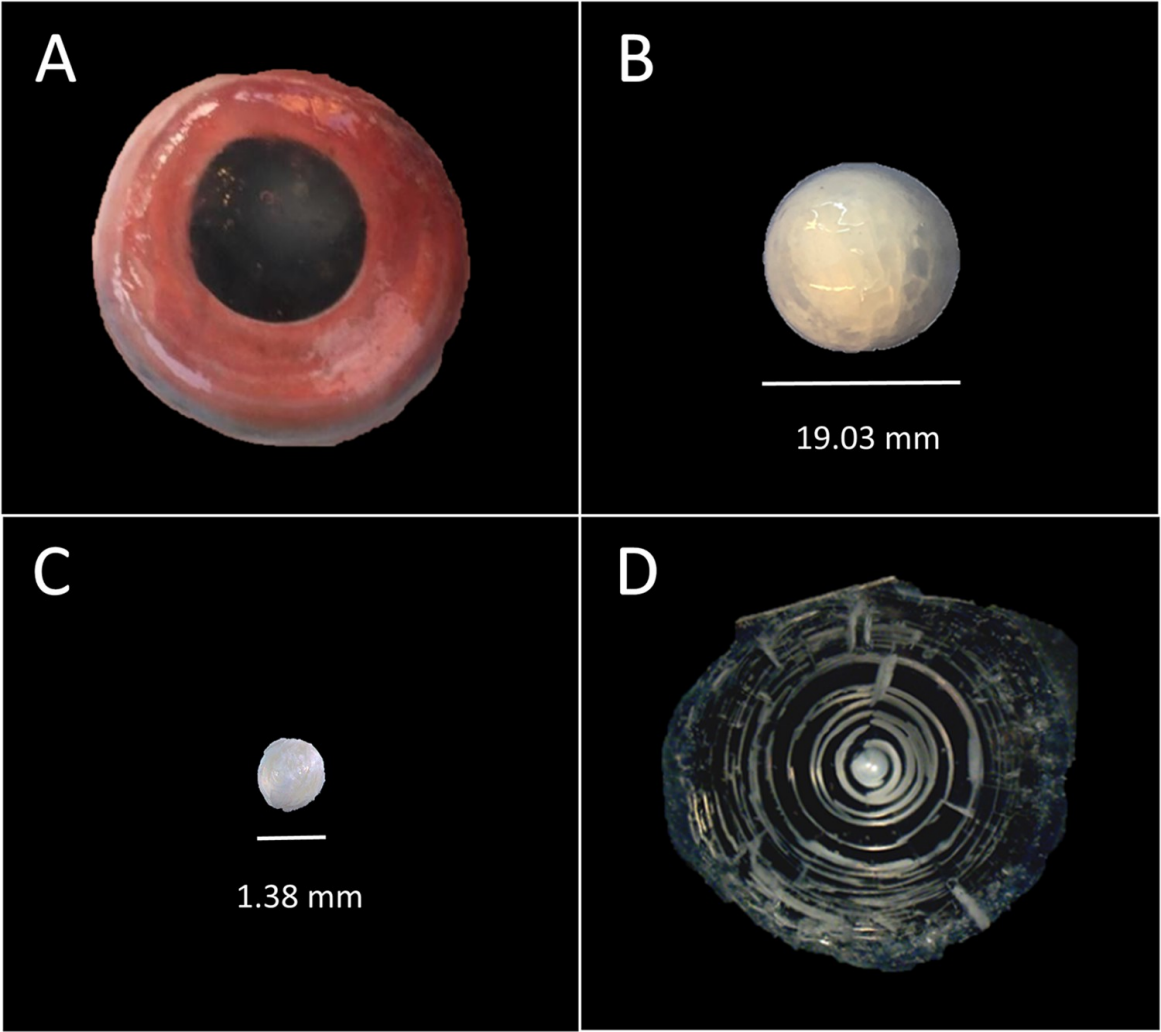
The process of extracting an eye lens core from a queen snapper left eye sample. (A) a queen snapper eye was extracted from a 708 mm FL fish, (B) an eye lens extracted from the thawed eye in image A (diameter = 19.03 mm), (C) the dried eye lens core (diameter = 1.38 mm) extracted from the eye lens in image B, and (D) the corresponding right eye lens sectioned via isomet saw, displaying the eye lens lamellae (concentric rings) and eye lens core. Reprinted under a CC BY license, with permission from Katherine Overly and Virginia Shervette, original copyright 2021.

To establish the target diameters of the eyes lens core region that represented approximately the first year of life for each species, we followed the methods from Shervette and Rivera Hernández [30]. This resulted in a target eye lens core diameter of 1.9 mm for blackfin snapper and 1.5 mm for queen snapper. Eyes from queen and blackfin snapper samples remained frozen until ready to process for lens core removal, which was initiated by thawing eyes at room temperature. Forceps and glassware were dipped in 10% HCL acid, rinsed with ultra- pure water, dried, and then baked in a muffle furnace at 550°C for a minimum of 6 hr. Using sterile forceps, each lens was removed from its eye. Lenses were placed in glass petri dishes and allowed to dry completely (minimum of 24 hr). Eye lens were peeled until reaching the species-specific target lens core diameter (Fig 3). The lens core for each sample was weighed to 0.1 mg and placed in a pre-treated glass vial for shipment to the National Ocean Sciences Accelerator Mass Spectrometry (NOSAMS) facility at Woods Hole Oceanographic Institute (WHOI) for Δ^14^C analysis.

Extraction of otolith cores followed the methods in Shervette et al. [31]. Otoliths used for core material Δ^14^C analysis were cleaned using a 10% bleach solution, rinsed with distilled water, allowed to air dry for 24 hr and embedded in epoxy resin. Following a 48 hr minimum cure time, the core area of the embedded otolith was extracted by cutting a transverse section at a width of 1.5 mm that contained the core, then mounted to a glass slide. Slides were affixed to a milling stage using paraffin wax, positioned and locked into place. The targeted core area included the region from the otolith primordium to the edge of the first opaque zone to ensure enough material was collected for a robust sample (minimum of 1.0 mg for high-resolution Δ^14^C values). Otolith core material for each sample was extracted using a computerized micromill instrument following the channel method of Shervette et al. [13]. The extracted otolith core material was weighed to the nearest 0.01 mg and stored in an acid-leached glass vial.

### Bomb radiocarbon analysis

Robust precision results (< 3.5‰ standard deviation [s.d.]) for Δ^14^C via AMS typically requires ≥ 0.1 mg of carbon for analysis. Fish otoliths are approximately 12% carbon by mass, which means to obtain Δ^14^C results with robust precision, a minimum of 1 mg of otolith core material is required. Extracting a minimum of 1 mg of otolith core material from species with small and fragile otoliths can be extremely difficult and requires specialized computerized micromilling systems. Eye lens cores, comprised of crystallin proteins, are approximately 50% carbon by mass [39, 40], which means for robust Δ^14^C results, a minimum of 0.3 mg of lens core material is required. All eye lens cores for queen snapper and blackfin snapper well exceeded the minimum mass requirement. Eye lens core and otolith core samples were analyzed for Δ^14^C and δ^13^C with accelerator mass spectrometry (AMS) at the NOSAMS WHOI. Sample processing and analysis protocols followed NOSAMS standard methods (http://www.whoi.edu/nosams/radiocarbon-data-calculations).) Reported values incorporate statistical and analytical sources of error in Δ^14^C measurements.

Queen and blackfin snapper Δ^14^C and corresponding estimated birth year results were overlaid on the north Caribbean reference Δ^14^C time series [13]. The estimated birth year of a sample equaled the year of collection minus the otolith increment. Peak spawning of queen snapper in the north Caribbean occurs in September (S1 Fig); therefore, the original birth year estimates for queen snapper samples were adjusted by adding 1.25 (midpoint in time that the core material of otoliths and lenses formed, assuming fish hatched on 1 September: 0.50 + 0.75). For example, a queen snapper with an estimated age of 10 y caught in 2020 would have an estimated integer birth year of 2010 plus 0.75 y to account for the 1 September hatch date, plus 0.50 y to account for the midpoint of the first year of life. This equals an adjusted fractional year of formation value of 2011.25 for the lens core or otolith core sample. Peak spawning for blackfin snapper in the north Caribbean occurs October–December [25], therefore the original birth year estimates for blackfin snapper were adjusted by adding 1.33 (midpoint in time that the core material of otoliths and lenses formed, assuming fish hatched on 1 November: 0.50 + 0.83).

Age estimation accuracy was evaluated by applying the method of Kastelle et al. [18]. This method examines potential ageing bias by purposely shifting birth year estimates ± 1–3 y, computing the sum of squared residuals (SSR) for eye lens/otolith core Δ^14^C and Δ^14^C predicted from the reference time series, and then comparing SSR estimates among shifted birth year models and the null model (i.e., birth year based on original age estimate). The model with the lowest SSR is considered the most parsimonious prediction of birth year [18]. If the null model produces the lowest SSR, then the age estimation method is inferred to be accurate.

### Depth versus size and age

To explore potential queen snapper ontogenetic shifts with depth of habitat, the relationships between fish length versus depth of capture and fish age versus depth of capture were examined using separate Kendall rank correlation coefficient analyses. Fish length data were collected as part of the current validation study, and age data from a more comprehensive set of queen snapper sample collections from the U.S. Caribbean [28]. An additional Kendall rank correlation coefficient analysis was computed to explore the potential relationship in blackfin snapper depth of capture and length for samples examined as part of the current study.

Larval size and daily age data collected by D’Alessandro et al. [26] were examined in relation to depth of collection. D’Alessandro et al. [26] randomly selected queen snapper from all peak spawning months, and from 1 mm standard length (SL) size bins to encompass the full size range of sample collections. To obtain daily age estimates as part of the larval study, one otolith from each selected fish was glued to a glass side, and an otolith section was obtained through polishing and otolith sections were aged [26]. In the current study, the relationships between depth and daily age, and between depth and fish length were evaluated using separate Kendall rank correlation coefficient analyses. For the independent variable of depth, the midpoint of each depth bin was used because the original study design [26] trawled for larval fish collections within specified depth bin ranges, rather than at consistent, specific depths.

## Results

### Bomb radiocarbon age estimation validation

**Fig 4:**
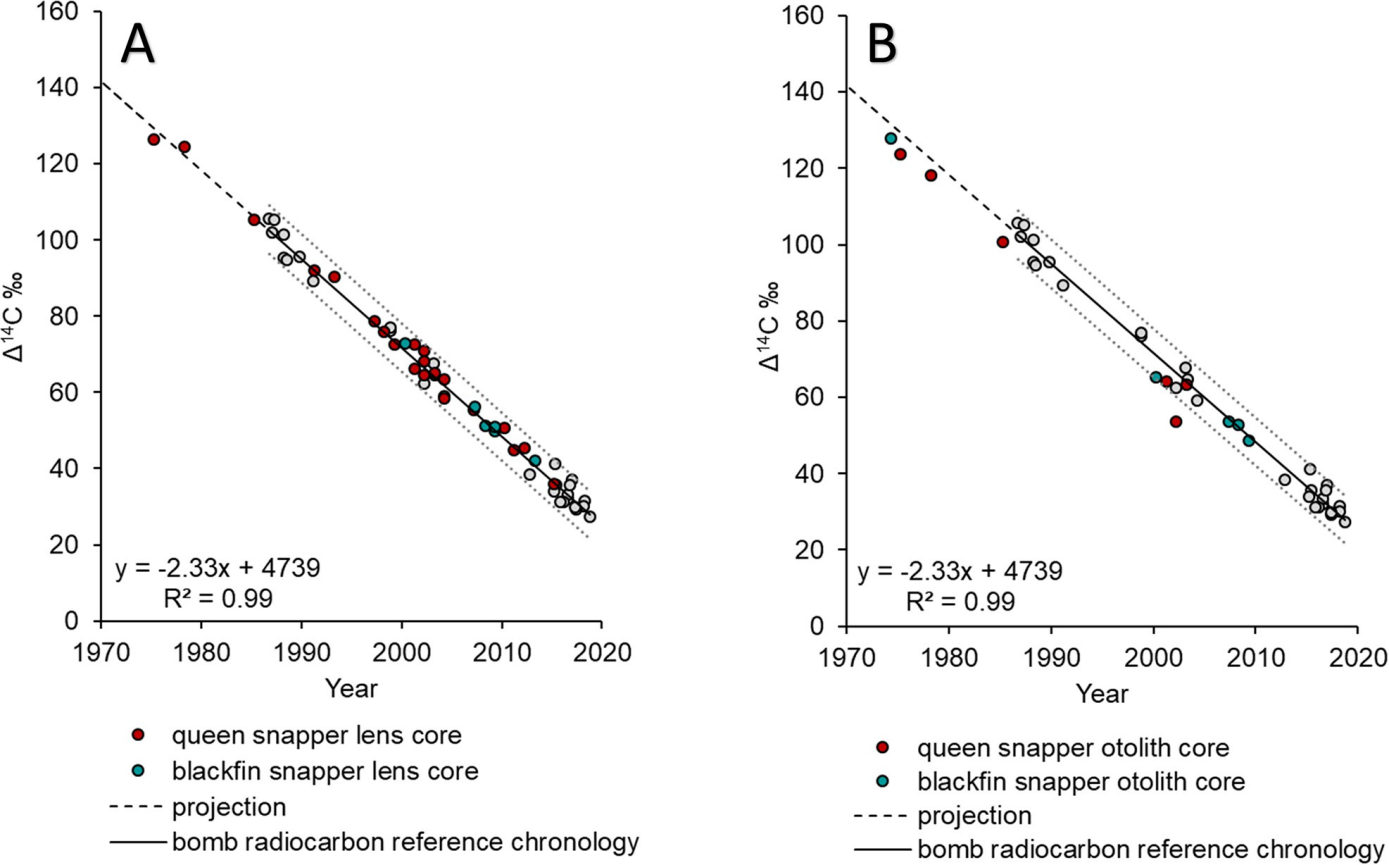
Results of Δ^14^C analysis via AMS. Δ^14^C values versus year of formation for, (A) queen snapper eye lens cores (n = 21), and blackfin snapper eye lens cores (n = 7), and (B) queen snapper otolith cores (n = 6), and blackfin snapper otolith cores (n = 5). The reference chronology linear regression (gray line) ± 95% PIs (dotted gray lines) (y = 4,680–2.304x; R^2^ = 0.99) plotted. Years include the decline period from 1987 to 2019 for U.S. Caribbean known-age red hind otolith Δ^14^C reference chronology (gray circles) from Shervette et al. [13], and the slope of the reference series projected back to 1974 (dashed black line) for visualization purposes.

**Fig 5:**
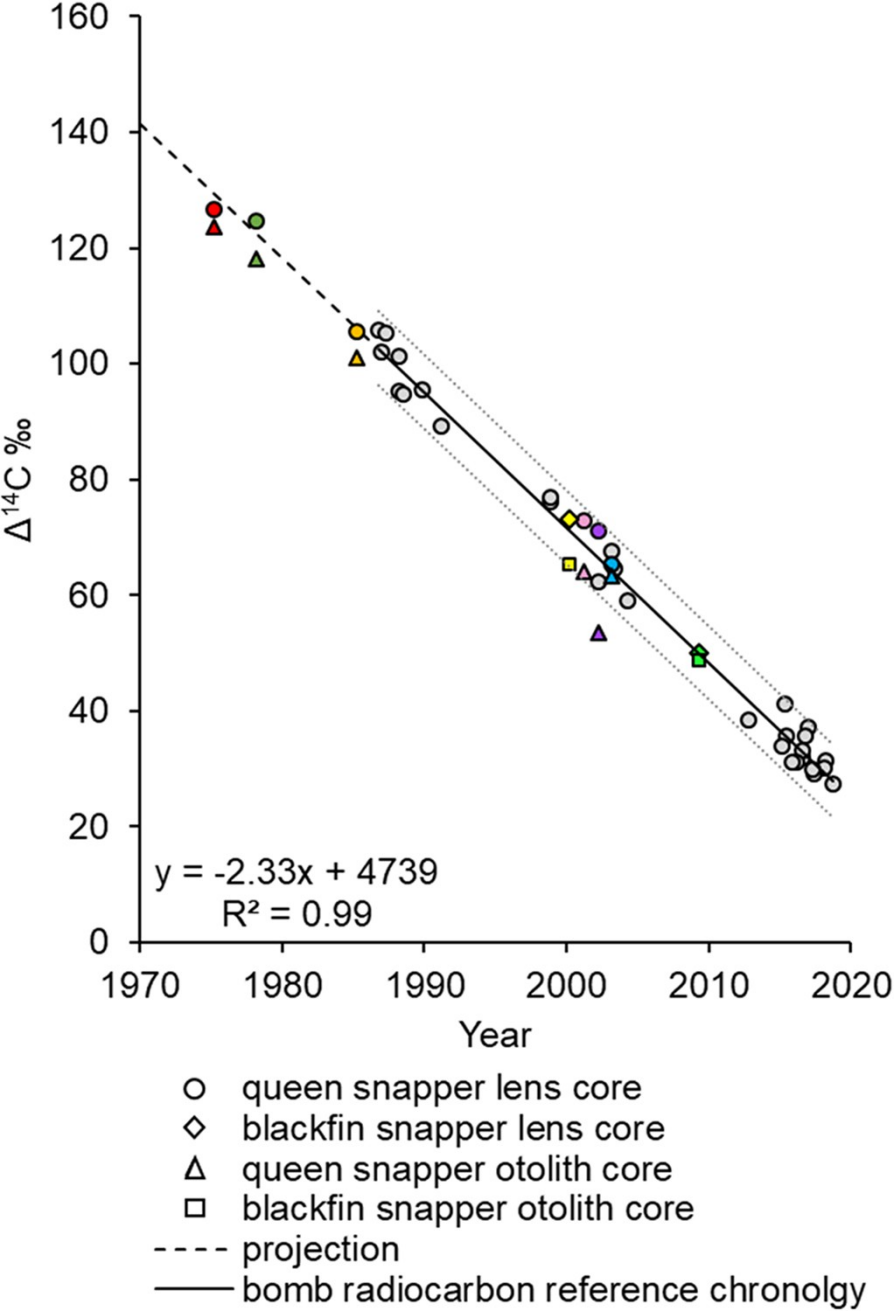
Comparison of Δ^14^C AMS analysis results between paired otolith core and eye lens core samples. Scatterplot of Δ^14^C values versus year of formation for queen snapper eye lens cores (red, orange, teal, pink, purple, blue circle; n = 6) with corresponding otolith core sample (red, orange, teal, pink, purple, blue triangle; n = 6), and blackfin snapper eye lens cores (yellow, green triangle; n = 2) with corresponding otolith core sample (yellow, green square; n = 2). The reference chronology linear regression (gray line) ± 95% PIs (dotted gray lines) (y = 4,680–2.304x; R^2^ = 0.99) plotted. Years include the decline period from 1987 to 2019 for U.S. Caribbean known-age red hind otolith Δ^14^C reference chronology (gray circles) from Shervette et al. [13], and the slope of the reference series projected back to 1974 (dashed black line) for visualization purposes.

For queen snapper, Δ^14^C eye lens core results were obtained from 21 samples with an estimated age range of 5–45 y and a corresponding birth year range of 1975–2015 (Table 1). Ten otolith samples were initially prepared for otolith core extraction; however, due to the fragile nature of queen snapper otoliths, four of the selected samples fractured during the milling process. This resulted in large chips external to the targeted otolith core contaminating the sample and ultimately rendering the material unsuitable for Δ^14^C analysis. Six queen snapper samples had paired lens core and otolith core Δ^14^C results; otolith core Δ^14^C values were consistently depleted for each sample compared to the lens core Δ^14^C results (Table 1; Figs 4 and 5). The eye lens core–otolith core Δ^14^C deviation ranged from 1.98 to 17.45‰.

**Table 1:** Queen snapper and blackfin snapper otolith core and eye lens core Δ^14^C age validation samples.

				Age estimate		Eye lens core data			Otolith core data		
Fish ID	NOSAMS ID	Species	Sample Year	Age (y)	Core YOF	Δ^14^C ‰	σ	δ^13^C ‰	Δ^14^C ‰	σ	δ^13^C ‰
ETOC01PR	164609	ETOC	2019	45	1975.25	126.45	2.20	-17.83	123.62	5.40	-6.30
ETOC02PR	164610	ETOC	2019	5	2015.25	36.05	2.00	-18.5	NR	NR	NR
ETOC03PR	165998	ETOC	2019	16	2004.25	58.57	2.20	-18.46	NR	NR	NR
ETOC04PR	165999	ETOC	2019	13	2007.25	55.33	2.10	-18.23	NR	NR	NR
ETOC05PR	166000	ETOC	2018	7	2012.25	45.37	2.40	-18.32	NR	NR	NR
ETOC06PR	166001	ETOC	2018	9	2010.25	50.8	2.60	-18.44	NR	NR	NR
ETOC07PR	166102	ETOC	2018	8	2011.25	44.77	2.20	-18.17	NR	NR	NR
ETOC08PR	166103	ETOC	2018	17	2002.25	64.62	2.10	-17.98	NR	NR	NR
ETOC09PR	166104	ETOC	2018	17	2002.25	70.98	2.30	-17.93	53.53	2.40	-4.77
ETOC10PR	166105	ETOC	2018	26	1993.25	90.24	2.20	-18.00	NR	NR	NR
ETOC11PR	166106	ETOC	2018	18	2001.25	66.18	2.10	-18.01	NR	NR	NR
ETOC12PR	166107	ETOC	2018	15	2004.25	63.31	2.20	-19.11	NR	NR	NR
ETOC13PR	166108	ETOC	2019	21	1999.25	72.55	2.10	-18.4	NR	NR	NR
ETOC14PR	166109	ETOC	2019	18	2002.25	68.17	2.20	-17.66	NR	NR	NR
ETOC15PR	166835	ETOC	2020	36	1985.25	105.44	2.40	-17.63	100.81	4.60	-6.04
ETOC16PR	166836	ETOC	2020	23	1998.25	75.96	2.80	NR	NR	NR	NR
ETOC17PR	166837	ETOC	2020	30	1991.25	92.15	2.30	-17.72	NR	NR	NR
ETOC18PR	166838	ETOC	2020	20	2001.25	72.73	2.40	-18.35	64.07	5.30	-5.54
ETOC19PR	166839	ETOC	2020	18	2003.25	65.16	2.30	-18.21	63.18	4.50	-6.52
ETOC20PR	166840	ETOC	2020	24	1997.25	78.59	2.30	-17.88	NR	NR	NR
ETOC21PR	167899	ETOC	2020	43	1978.25	124.47	2.90	-17.73	118.14	5.00	-5.50
LUBU01PR	165846	LUBU	2018	19	2000.33	72.96	2.20	-17.7	65.28	6.20	-5.67
LUBU02PR	165844	LUBU	2019	10	2009.33	49.9	2.20	-17.96	48.69	2.10	NR
LUBU03PR	159494	LUBU	2019	20	2000.33	68.26	2.10	-17.50	NR	NR	NR
LUBU01STT	165841	LUBU	2020	13	2008.33	51.3	2.20	-17.45	NR	NR	NR
LUBU02STT	165842	LUBU	2020	14	2007.33	56.17	3.00	-16.86	NR	NR	NR
LUBU03STT	165843	LUBU	2019	11	2009.33	50.88	2.20	-17.95	NR	NR	NR
LUBU04STT	165845	LUBU	2020	8	2013.33	42.15	2.20	-17.08	NR	NR	NR
LUBU05STT	165345	LUBU	2018	11	2008.33	NR	NR	NR	52.81	2.10	-4.26
LUBU06STT	165344	LUBU	2016	43	1974.33	NR	NR	NR	127.76	2.30	NR
LUBU07STT	165347	LUBU	2018	12	2007.33	NR	NR	NR	53.66	2.20	NR

“Core YOF” equals sample year minus estimated ages. Adjusted year of formation values are the year of formation plus 1.25 and 1.33 y (queen, blackfin, respectively); see text for rationale. YOF = year of formation, NR = not reported, ETOC = queen snapper, LUBU = blackfin snapper, y = year.

For blackfin snapper, Δ^14^C results were obtained for a total of ten samples with an estimated age range of 8–43 y and a corresponding birth year range of 1974–2013 (Table 1; paired eye lens core—otolith core samples n = 2; only eye lens core samples n = 5; only otolith core samples n = 3). For the two samples with paired eye lens core and otolith core Δ^14^C results, otolith core Δ^14^C values did not consistently differ from eye lens cores by large values. For the eye lens cores, otolith core Δ^14^C deviations for the two samples were 1.21 and 7.68‰ (Table 1; Fig 4). Blackfin snapper otoliths are large and dense compared to queen snapper; as a result, no issues with fracturing occurred during the milling process.

Bomb radiocarbon results ranged from 36.05–126.45‰ for queen snapper, and 42.15–127.76‰ for blackfin snapper. All queen snapper and blackfin snapper otolith opaque zone count birth year estimates had eye lens and otolith core Δ^14^C values that fell within the 95% PIs around the linear regression fit to the north Caribbean reference series (Fig 4), apart from one sample (the queen snapper otolith core sample with the depleted difference of -17.45‰ compared to the paired lens core Δ^14^C value from the same fish).

Results from the ageing bias analyses of queen and blackfin snappers indicated that birth year estimates derived from sagittal otolith thin section increment counts were accurate. For the 21 queen snapper samples with eye lens core Δ^14^C results, the original age estimates (null model) had the lowest SSR (93), while the purposefully biased age estimates resulted in SSR values ranging from 174 (-1 y model) to 1188 (+3 y model; Table 2). Similarly, results from the ageing bias analysis for the seven blackfin snapper samples with eye lens core Δ^14^C results indicated that the original age estimates had the lowest SSR (16), while SSR ranged from 55 (-1 y model) to 414 (+3 y model) among the purposefully biases age estimate models (Table 2).

**Table 2:** Results from ageing bias analysis.

		Queen snapper SSR		Blackfin snapper SSR	
Age Model	Bias Applied (y)	Eye lens	Otolith	Eye lens	Combined
		N = 21	N = 6	N = 7	N = 10
Null	0	93	270	16	29
-1	-1	174	457	55	106
-2	-2	478	708	170	289
-3	-3	1005	1023	358	578
+3	+3	1188	90	414	436
+2	+2	600	87	208	194
+1	+1	235	147	76	59

Sum of Square Residuals (SSR) ageing bias analysis results of queen snapper and blackfin snapper. Birth year estimates were biased by +/- 1 to 3 years (y) for the species and the squared residuals from the predicted reference Δ^14^C reference chronology were computed.

As queen snapper habitat preference during early life remains undocumented and otolith cores were exceedingly difficult to precisely extract due to the small and fragile nature of queen snapper otoliths, we hypothesized that Δ^14^C would be depleted in otolith cores versus lens cores and thus bias otolith core SSR results. Therefore, separate SSR ageing bias analyses were computed for queen snapper otolith core Δ^14^C sample results. The lowest SSR for the queen snapper otolith core Δ^14^C ageing bias analysis was +2 y (SSR = 87) while SSR ranged from 90 to 1023 for the null and shifted birth year models (Table 2). Alternatively, as blackfin snapper are known to inhabit shallow waters within the reefscape as juveniles [24, 25], we combined Δ^14^C results for the seven blackfin snapper eye lens core samples with the three otolith core results for only the additional ageing error analysis. The lowest SSR for blackfin snapper combined samples (n = 10) was the null model (SSR = 29), while SSR ranged from 59 to 578 among the purposefully biased age estimate models (Table 2).

### Depth versus size and age

**Fig 6:**
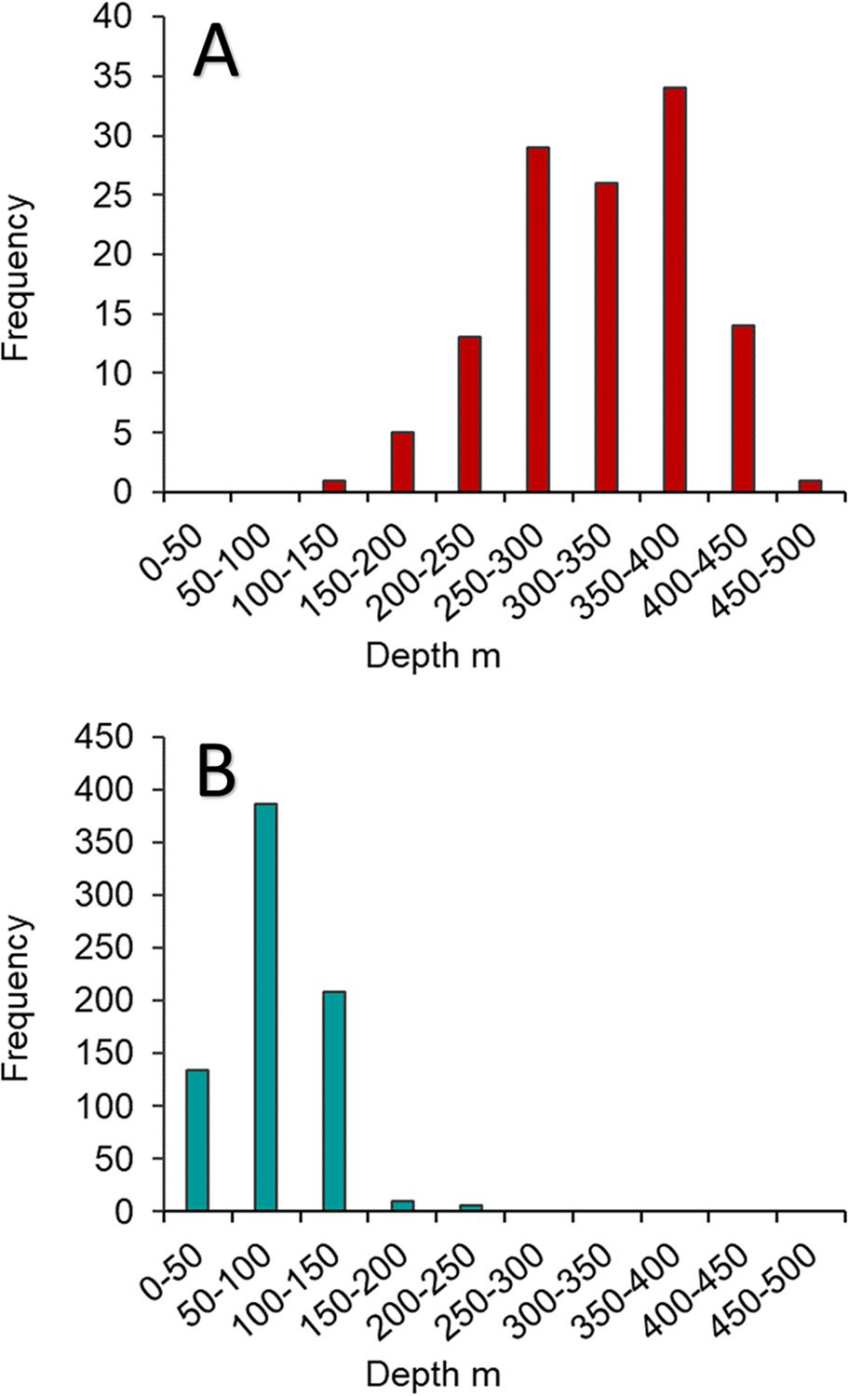
Depth distribution of queen snapper and blackfin snapper. Frequency of (A) queen snapper caught during fishery-independent sampling at depths of 100–500 m, by 50 m depth bins (n = 123); and (B) blackfin snapper caught during fishery-independent sampling at depths of 10–500 m, by 50 m depth bins (n = 763).

**Fig 7:**
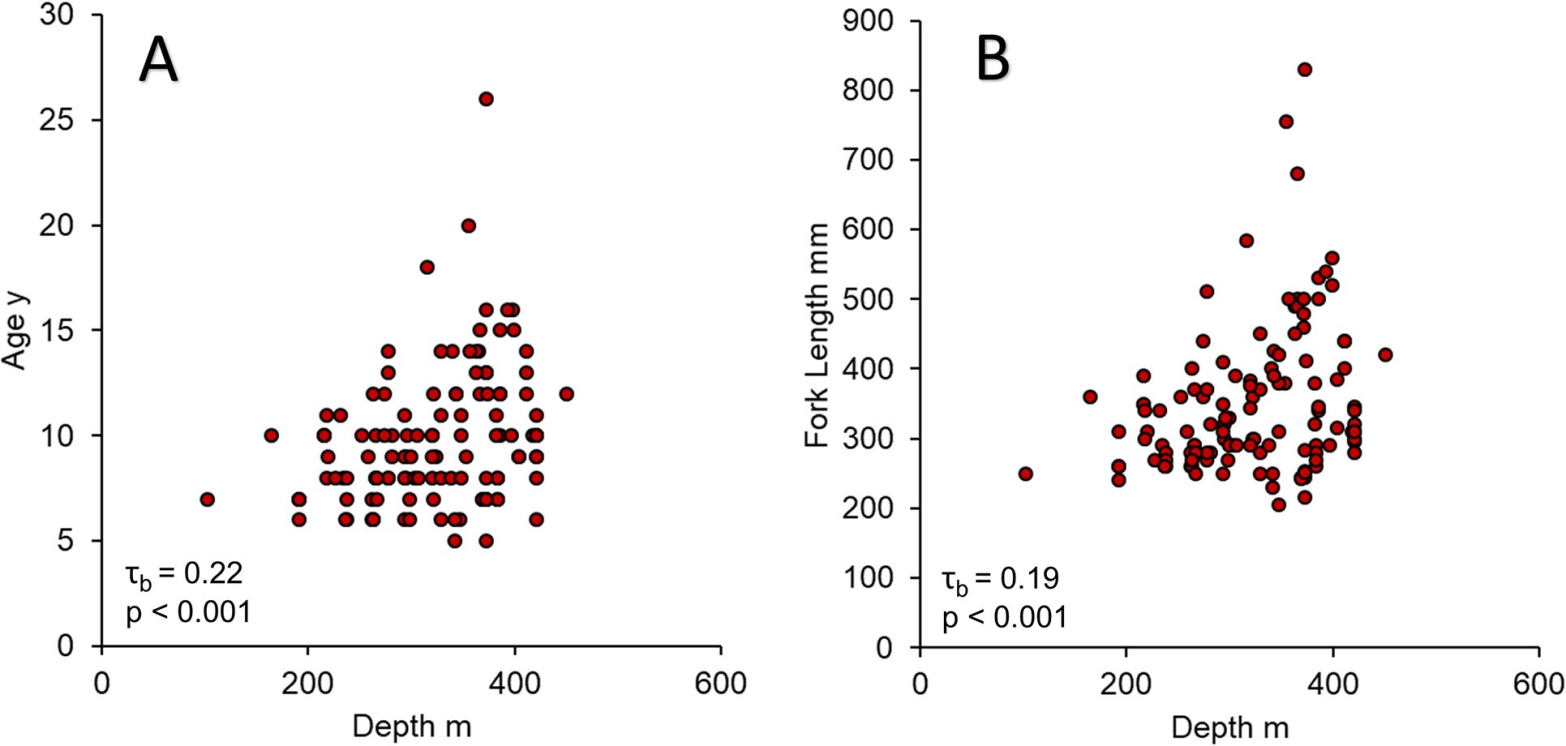
Queen snapper depth distribution by fork length and age. Queen snapper depth distribution from fishery-independent sampling in Puerto Rico plotted by (A) fork length (mm) and, (B) age (y). The Kendall rank correlation coefficients (τ_b_) are denoted in the graphs.

**Fig 8:**
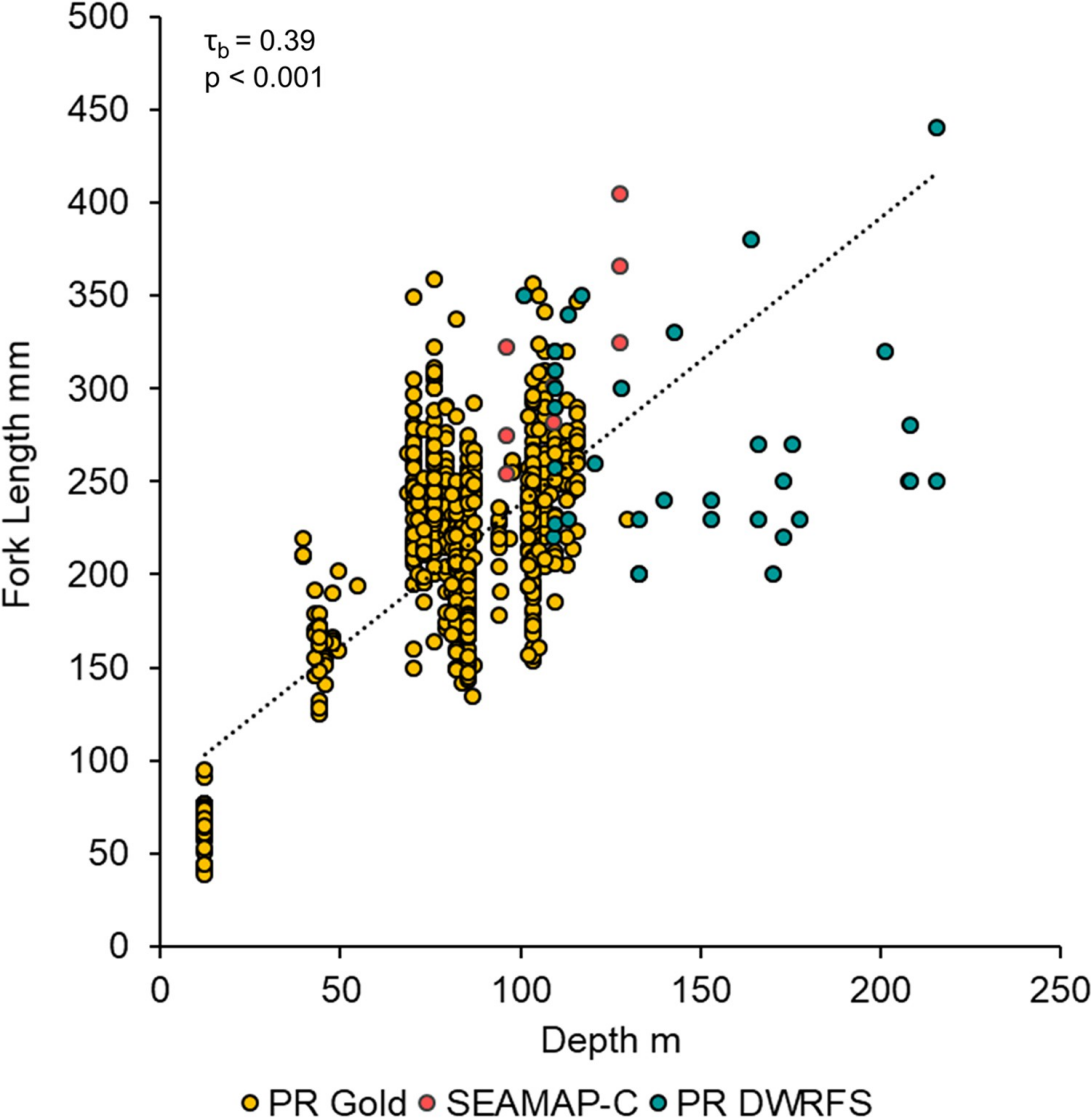
Blackfin snapper depth distribution by fork length. Blackfin snapper depth distribution from fishery-independent sampling in Puerto Rico plotted by fork length (mm) versus depth (m). The Kendall rank correlation coefficient (τ_b_) for the combined data is τ_b_ = 0.39, p < 0.001.

Queen snapper (n = 123) FI PR samples were collected from depths of 102–451 m (Fig 6). Queen snapper age and length were significantly positively correlated with depth (Fig 7); correlations were moderate with respect to age (τ_b_ = 0.22, p < 0.001), and weak with respect to length (τ_b_ = 0.19, p < 0.001). Small, young fish were found across the depth range sampled, although larger (> 300 mm FL) and older (> 10 y) fish were mostly found deeper than 300 m. Blackfin snapper (n = 744) FI PR samples were collected from depths of 12–215 m (Fig 6). Blackfin snapper length was significantly positively correlated with depth (Fig 8), and correlations were strong (τ_b_ = 0.39, p < 0.001).

**Fig 9:**
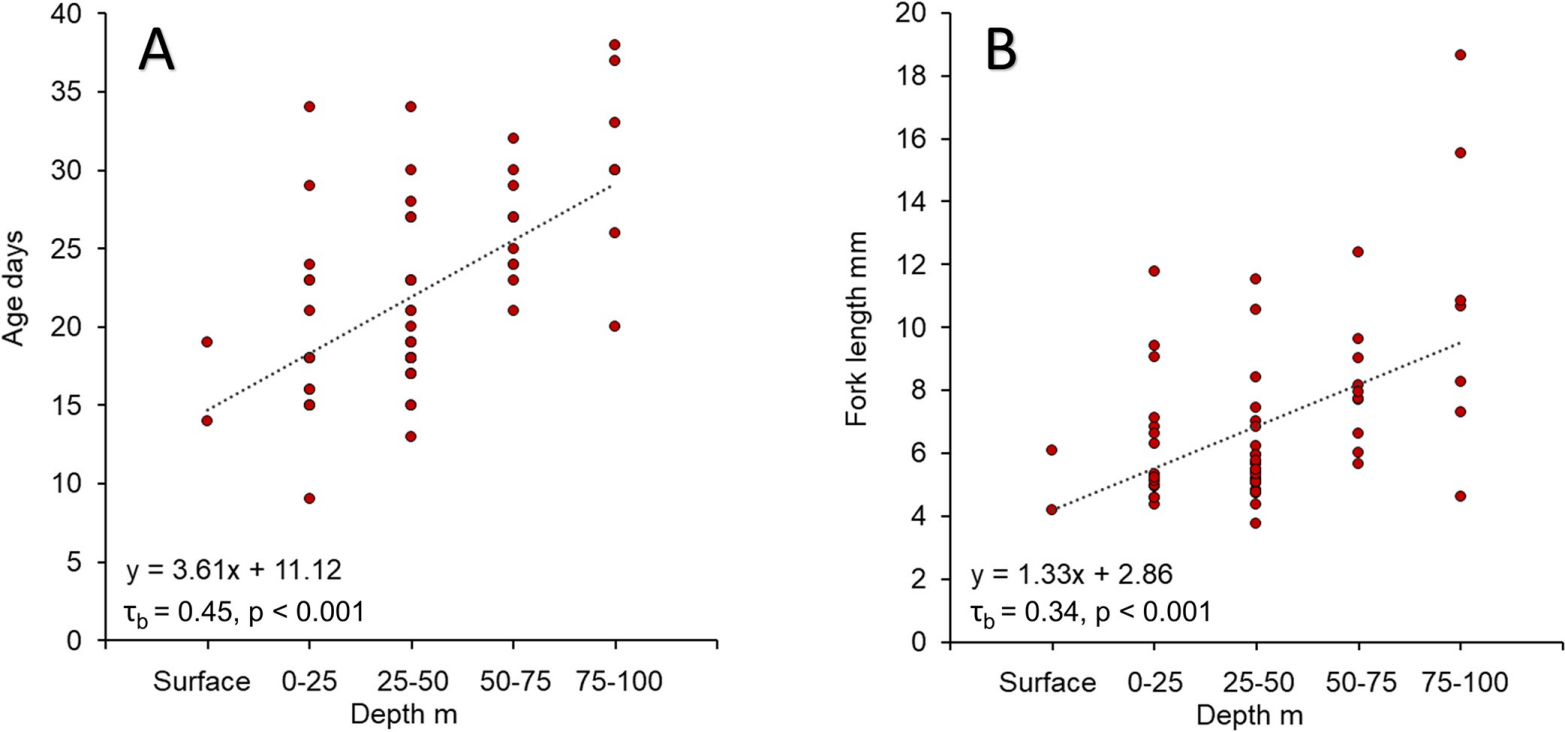
Larval queen snapper depth distribution by fork length and age. Larval queen snapper depth distribution from fishery-independent sampling in the Straits of Florida plotted by (A) age in days (day) versus depth in meters (m), and (B) fork length (mm) versus depth (m). The Kendall rank correlation coefficient (τ_b_) are denoted in the graphs.

Larval queen snapper collected from depths of 0–100 m were aged by D’Alessandro et al. [26]. Larval queen snapper (n = 61) FL (mm) and daily age were significantly positively correlated with depth (Fig 9). Correlations were relatively strong with depth for age (τ_b_ = 0.45, p < 0.001), and length (r = 0.34, p < 0.001).

## Discussion

The current study is the first to successfully validate age estimation for queen snapper and blackfin snapper while increasing knowledge of their longevities; both species can attain maximum ages greater than 40 y. Application of Δ^14^C age estimation validation demonstrated otolith increments are formed annually in queen snapper and blackfin snapper otoliths and can be counted to estimate ages accurately. Additionally, this study is the first to exclusively utilize eye lens cores to validate age estimates for a tropical deepwater species whose juvenile life history is unknown. The current study also indicates that previous estimates of longevity for queen and blackfin snappers were low (8 y and 27 y, respectively) [41, 42].

### Species longevity

Although the oldest queen snapper directly validated in the current study was 45 y, the validated ageing method used in this study documented several older queen snapper from the GoM, including a maximum age of 63 y [28]. Thus, estimated longevity for queen snapper was extended by over 50 y from the previous estimate of 8 y [42–44]. For blackfin snapper, the current study documented a new maximum age of 43 y, an increase from the previously reported maximum age of 27 y [41]. Maximum ages of > 50 y have been documented in eteline snappers from the Indo-Pacific [6, 7, 45, 46] and > 40 y for lutjanids [47], thus the maximum ages observed in the current study are consistent with longevity estimates for congeners elsewhere.

Several studies have documented latitudinal gradients related to maximum size and age for ectotherms [48–52]. Maximum size and age in many fishes increase with increasing latitude and decreasing temperature [49–56], whereas growth and mortality rates generally decrease with increasing latitude and decreasing temperature [57–59]. For example, a study conducted by Lowe et al. [48] found that the two-spined angelfish *Centropyge bispinosa*, a tropical reef fish species, exhibited regional variation in maximum age that correlated with latitude and water temperature. Regional variation in maximum age and size is important when considering our findings, as queen and blackfin snappers occur throughout the western Atlantic from North Carolina to Brazil and throughout the GoM. This distribution range includes many of the Caribbean islands such as PR and the U.S. Virgin Islands (USVI) and covers a broad range of latitudes. It is plausible then that life history parameters, such as longevity, vary regionally for the two species within their geographic range. Evidence from a concurrent study examining population demographics of queen snapper across the U.S. Caribbean and GoM suggests that queen snapper may attain an older maximum age in the GoM compared to the U.S. Caribbean [28].

Blackfin snapper also appears to exhibit regional patterns in maximum size and age. As part of a concurrent study investigating latitudinal differences in size, age, and growth for blackfin snapper, we have estimated ages for over 3,000 samples collected from three main regions of the western Atlantic: U.S. Caribbean waters; waters of North Carolina and South Carolina, USA (NC/SC); and waters of Belize and Honduras. The maximum validated age from the current study (43 y) extended longevity for blackfin snapper by 16 y. Previously, the reported maximum age was 27 y for blackfin snapper samples collected from waters off the southeastern U.S. (North Carolina through Florida) combined with samples from the U.S. Caribbean [41]. The 43 y fish in the current study was a medium-sized individual (405 mm FL/443 mm TL) caught via hook and line during a FI survey from a protected area south of St. Thomas, USVI, at a depth of 127 m. The largest blackfin snapper we have aged (705 mm FL/855 mm TL) was caught in waters of Honduras at a depth of 140 m during collections for Baremore et al. [60], which had an estimated age of 38 y. In contrast, the largest blackfin snapper we have aged from the U.S. Caribbean was 466 mm FL/504 mm TL and caught by a commercial fisher in southwest PR, which had an estimated age of 28 y. Burton et al. [41] reported on age estimates for blackfin snapper from North Carolina through Florida and U.S. Caribbean waters (n = 622) and noted that 1% of fish in that study attained ages > 15 y. Blackfin snapper appears to have a relatively fast growth rate, and obtained a mean observed length of 372 mm TL by 4 y, with growth rate slowing after a mean observed length of 473 mm TL by age 7 [41]. This seems to indicate that although blackfin snapper can attain ages greater than 15 y, older fish may be extremely rare.

### Bomb radiocarbon and depth

The earliest growth of eye lens material as a conserved radiocarbon reference point that represents environmental levels for birth year was recently demonstrated for deepwater elasmobranchs and subtropical/tropical teleost fishes [29–32, 61]. The general agreement between otolith cores and eye lens cores for shallow water species is fortuitous by providing an alternative to the use of otoliths cores. However, the agreement may be dependent on the early life history of the species (specifically habitat depth of juveniles) with eye lenses remaining reflective of the mixed surface layer Δ^14^C values, while otolith cores may not. In the current study, we found that queen snapper otolith core Δ^14^C values were consistently depleted compared to eye lens core Δ^14^C values. These results may be due to a combination of factors, including a depleted amount of Δ^14^C in the otolith core owing to juvenile queen snapper residing in deeper waters, or processing issues related to the difficulty in extracting solely core material that represented the first year of life because queen snapper otoliths are characteristically small, fragile, and prone to chipping during the handling process. The consistent depleted Δ^14^C results from queen snapper otolith cores compared to eye lens cores in our study (n = 6) may indicate that juvenile queen snapper spend a portion of their first year of life at water depths > 200 m in which Δ^14^C DIC is depleted relative to surface waters [62–64]. Andrews *et al*. [4] also utilized Δ^14^C to evaluate ageing accuracy of an *Etelis* species. Their study documented that for the majority of the otolith core samples analyzed for Onaga *Etelis coruscans* (n = 7), Δ^14^C values were marginally depleted relative to the estimated age of individuals for fish with birth years during the rapid rise period of the Δ^14^C reference time series. This could be the result of consistent under-ageing or processing issues negatively affecting precise extraction of otolith core material. However, when combined with results for queen snapper in the current study, the seemingly depleted otolith core Δ^14^C values for Onaga in the Pacific support the possibility that some habitats used by juveniles of *Etelis* species may occur in deeper waters (> 200 m depth) characterized by depleted Δ^14^C.

Little is known about the habitat and depth preferences of juvenile queen snapper, although Gobert et al. [27] reported collecting several juveniles < 100 mm TL at depths greater than 300 m. Additional evidence from the analysis of depth versus size and age in the current study indicated that queen snapper do not appear to exhibit a strong ontogenetic shift in depth with size or age [28]. Data from D’Alessandro et al. [26] suggest larval queen snapper are present as deep as 100 m, but not caught at depths < 20 m; whereas other shallow-water Lutjanids (*Lutjanus apodus*, *L*. *analis*, *L*. *synagris*, *L*. *griseus*, *and Ocyurys chrysus*) were found at depths < 20 m. Additionally, D’Alessandro et al. [26] noted the only larger larval fishes caught in the trawls were those from the deep-dwelling taxa, including queen snapper. Gobert et al. [27] documented small (55–70 mm FL) individuals off Guadeloupe at 490 m. These studies provide evidence that early post settlement juveniles are not restricted to the shallowest part of the species depth range. Misa et al. [65] noted juvenile Onaga *E*. *coruscans* (150 mm FL) present on low relief hard bottom habitat in the Pacific at depths > 250 m, but the sample size was small (n = 2) and the study concluded habitat association could not be determined for juveniles. There is some evidence to support the possibility that *Etelis* snapper species have a longer pelagic duration than *Lutjanus* snappers; Leis [66] noted larval queen snapper are “pelagic” to at least 50 mm body length, but no depth information was associated with samples in that study.

If queen snapper exhibited a clear shift in depth with increasing size and age, then FI collections efforts in the shallower depths should have obtained a proportionally greater amount of smaller, younger fish. However, in the current study, queen snapper 100–425 mm FL were collected across the full depth range sampled (100–500 m). Notably, fish larger than 500 mm were absent at depths < 250 m (Fig 7). The increase in the proportion of fish larger than 500 mm FL with increased depth could be due to multiple factors. Larger fish may have been targeted by fishers at shallower depths but are now largely absent from 100–250 m. Preferred prey items or sufficient prey quantity for larger queen snapper may only exist in deeper waters. While robust diet data is not currently available for queen snapper, in a study conducted by Williams et al. [67] it was noted that stomach contents of larger fish (508–1016 mm FL) did differ compared to smaller fish (203–508 mm FL) although total sample size was small (n = 157). Stomach content specimens genetically identified as *Bonapartia pedaliota*, *Hypogeum benioiti*, *and Sigmops elongate*s were more commonly found in queen snapper > 508 mm FL, whereas *Diaphus brachycephalus*, *D*. *dumerilii*, *Elagatis bipinnulata*, *Lepidophanes guentheri*, and *Myctophum nitidulum* were identified in a larger number of smaller queen snapper (< 508 mm FL) [67]. Studies exploring depth, age, and diet composition of queen snapper are needed to further explore this hypothesis. Larger fish could have habitat requirements (as relates to physical water properties) that are only met in deeper parts of their depth range; more specifically, occupying deeper waters characterized by lower water temperatures leads to lower metabolic cost, increased longevity, and a greater allocation of energy to reproductive capabilities [68]. Currently, it is unclear what combination of these hypotheses best explains the depth pattern observed for queen snapper.

Blackfin snapper exhibit an ontogenetic shift in habitat with juveniles occurring at higher abundances in shallow waters of the shelf [24, 25] and larger adults utilizing habitat in deeper shelf waters, often occurring at the shelf edge in association with hardbottom and complex reef structure [25, 69–71]. Concurrent FI sampling in PR found blackfin snapper between depths of 60–216 m; therefore, we would expect blackfin eye lens core Δ^14^C values to reflect the same or similar value to the otolith core Δ^14^C values as demonstrated for red snapper *Lutjanus campechanus* [29].

### Usefulness of eye lenses

Previous Δ^14^C age validation efforts of reef fishes from GoM and the Caribbean have relied on the use of computerized micromilling systems to extract otolith core material to obtain birth year Δ^14^C [10, 13, 21, 72]. These systems are essential for accurately coring otoliths because they enable precise extraction of target material for AMS analysis. However, access to a computerized micromill is not easily attainable and potentially cost-prohibitive for small- to medium-sized research labs conducting reef fish life history work in the Caribbean. The fragility and small size of queen snapper otoliths made obtaining a sagittal otolith core sample for Δ^14^C analysis time consuming and subject to a high risk of contamination from younger regions of the otolith. The current study further demonstrated that eye lens cores provide an accessible and accurate alternative for age validation efforts of Caribbean reef fish species and are particularly useful for species that may reside in deeper waters as juveniles, or have small, fragile otoliths.

## Supporting information

S1 Fig Reproductive seasonality of female queen snapper. Preliminary gonad histology results for queen snapper female reproductive phase by month of sample collections from Puerto Rico sites.
(TIF)

S1 Table Supporting information for queen snapper and blackfin snapper analyzed for Δ^14^C with AMS. Specimens for which no standard length was recorded are shown as NR. Reported lengths are standard length (SL), fork length (FL), and total length (TL). Island platform where specimens were caught included Puerto Rico (PR) and St. Thomas (STT). g = grams, y = years.
(DOCX)
