# Supplementary figures and images for: Caribbean deepwater snappers: Application of the bomb radiocarbon age estimation validation in understanding aspects of ecology and life history
Source: PLoS One. 2023 Dec 27;18(12):e0295650. doi: 10.1371/journal.pone.0295650 (PMC10752517; doi:10.1371/journal.pone.0295650)

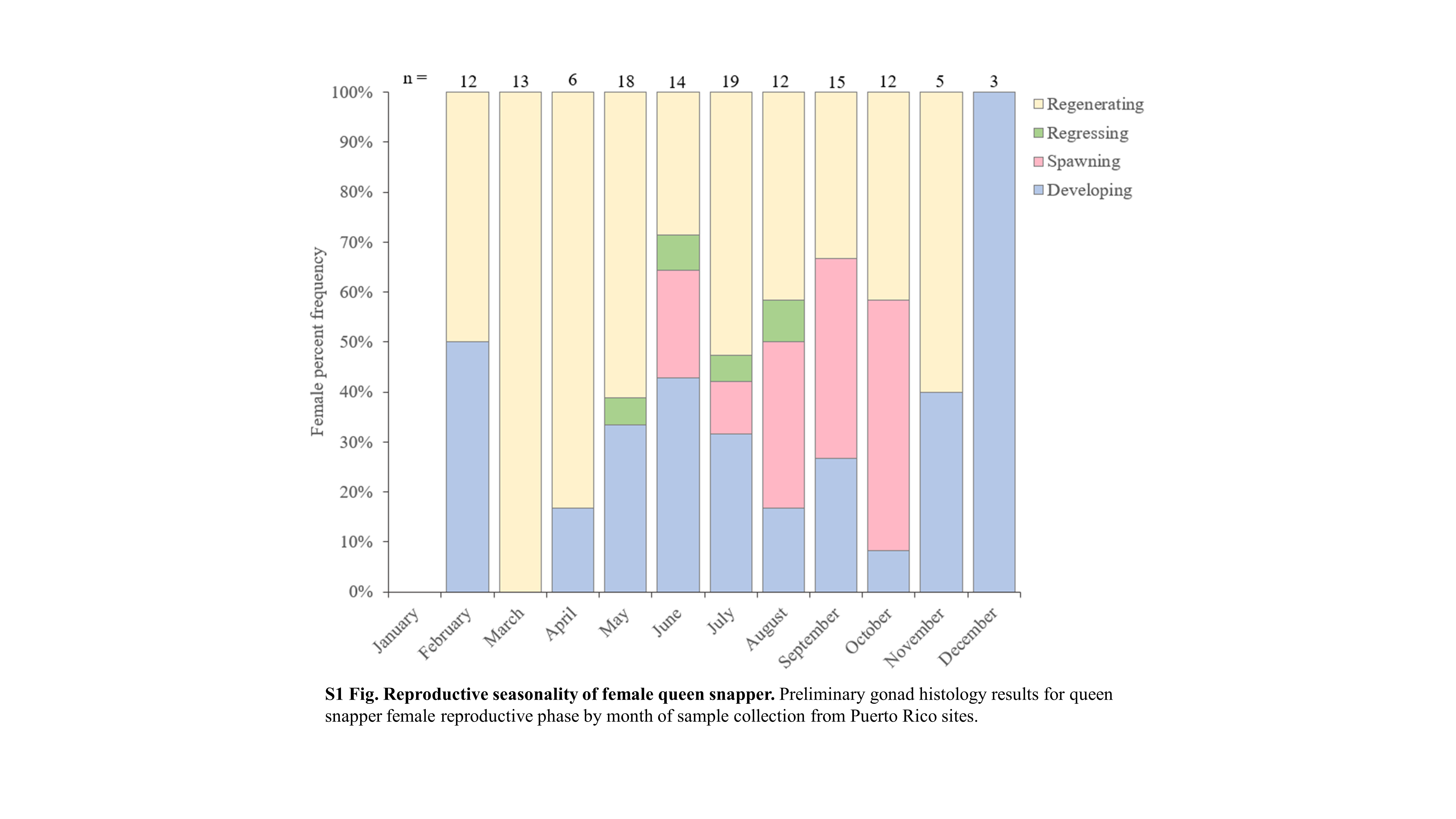

Supplement: S1 Fig — Preliminary gonad histology results for queen snapper female reproductive phase by month of sample collections from Puerto Rico sites. (TIF) [file pone.0295650.s001.tif]
