# Supplementary material for: Caribbean deepwater snappers: Application of the bomb radiocarbon age estimation validation in understanding aspects of ecology and life history
Source: PLoS One. 2023 Dec 27;18(12):e0295650. doi: 10.1371/journal.pone.0295650 (PMC10752517; doi:10.1371/journal.pone.0295650)
Supplement: S1 Table — Specimens for which no standard length was recorded are shown as NR. Reported lengths are standard length (SL), fork length (FL), and total length (TL). Island platform where specimens were caught included Puerto Rico (PR) and St. Thomas (STT). g = grams, y = years. (DOCX) [file pone.0295650.s002.docx]

**S1 Table.** **Supporting information for queen snapper and blackfin snapper analyzed for Δ^14^C with AMS.**

Specimens for which no standard length was recorded are shown as NR. Reported lengths are standard length (SL), fork length (FL), and total length (TL). Island platform where specimens were caught included Puerto Rico (PR) and St. Thomas (STT). g = grams, y = years.

| **Fish ID** | **Species** | **SL (mm)** | **FL (mm)** | **TL (mm)** | **Whole weight (g)** | **Island** | **Date collected** | **Age (y)** |
| --- | --- | --- | --- | --- | --- | --- | --- | --- |
| ETOC01PR | queen snapper | 650 | 708 | 863 | 5039 | PR | 7/19/2019 | 45 |
| ETOC02PR | queen snapper | 156 | 178 | 217 | 95 | PR | 6/3/2019 | 5 |
| ETOC03PR | queen snapper | 464 | 508 | 665 | 2030 | PR | 7/19/2019 | 16 |
| ETOC04PR | queen snapper | 434 | 478 | 607 | 1690 | PR | 7/19/2019 | 13 |
| ETOC05PR | queen snapper | 195 | 225 | 280 | 200 | PR | 11/6/2018 | 7 |
| ETOC06PR | queen snapper | 262 | 295 | 375 | 426 | PR | 11/6/2018 | 9 |
| ETOC07PR | queen snapper | 186 | 208 | 254 | 162 | PR | 11/6/2018 | 8 |
| ETOC08PR | queen snapper | 567 | 643 | 813 | 3570 | PR | 11/24/2018 | 17 |
| ETOC09PR | queen snapper | 563 | 623 | 798 | 3909 | PR | 11/24/2018 | 17 |
| ETOC10PR | queen snapper | 633 | 707 | 900 | 5020 | PR | 11/24/2018 | 26 |
| ETOC11PR | queen snapper | 567 | 621 | 799 | 3433 | PR | 11/24/2018 | 18 |
| ETOC12PR | queen snapper | 448 | 498 | 618 | 1877 | PR | 11/24/2018 | 15 |
| ETOC13PR | queen snapper | 595 | 661 | 930 | 4161 | PR | 7/18/2019 | 21 |
| ETOC14PR | queen snapper | 315 | 357 | 420 | 744 | PR | 7/18/2019 | 18 |
| ETOC15PR | queen snapper | 719 | 762 | 967 | 5726 | PR | 2/2/2020 | 36 |
| ETOC16PR | queen snapper | 660 | 680 | 871 | 4351 | PR | 2/4/2020 | 23 |
| ETOC17PR | queen snapper | 640 | 665 | 830 | 4391 | PR | 2/4/2020 | 30 |
| ETOC18PR | queen snapper | 575 | 597 | 711 | 3172 | PR | 2/4/2020 | 20 |
| ETOC19PR | queen snapper | 652 | 673 | 870 | 4565 | PR | 2/4/2020 | 18 |
| ETOC20PR | queen snapper | 750 | 800 | 964 | 6916 | PR | 2/2/2020 | 24 |
| ETOC21PR | queen snapper | 690 | 710 | 840 | 5074 | PR | 2/2/2020 | 43 |
| LUBU01PR | blackfin snapper | 365 | 437 | 485 | 1745 | PR | 6/18/2022 | 18 |
| LUBU02PR | blackfin snapper | NR | 440 | 470 | 1420 | PR | 5/28/2019 | 9 |
| **Fish ID** | **Species** | **SL (mm)** | **FL (mm)** | **TL (mm)** | **Whole weight (g)** | **Island** | **Date collected** | **Age (y)** |
| LUBU03PR | blackfin snapper | NR | 366 | 400 | 965 | PR | 1/17/2019 | 20 |
| LUBU01STT | blackfin snapper | 299 | 345 | 372 | 749 | STT | 2/12/2020 | 13 |
| LUBU02STT | blackfin snapper | 287 | 334 | 365 | 717 | STT | 2/10/2020 | 14 |
| LUBU03STT | blackfin snapper | 246 | 292 | 317 | 439 | STT | 8/20/2019 | 11 |
| LUBU04STT | blackfin snapper | 246 | 295 | 319 | 467 | STT | 1/12/2020 | 8 |
| LUBU05STT | blackfin snapper | NR | 345 | 375 | 695 | STT | 10/2/2018 | 11 |
| LUBU06STT | blackfin snapper | NR | 405 | 443 | 1125 | STT | 9/16/2016 | 43 |
| LUBU07STT | blackfin snapper | 359 | 420 | 452 | 1360 | STT | 9/19/2018 | 12 |
